# Assessing precision and requirements of three methods to estimate roe deer density

**DOI:** 10.1371/journal.pone.0222349

**Published:** 2019-10-10

**Authors:** Andrea Marcon, Daniele Battocchio, Marco Apollonio, Stefano Grignolio

**Affiliations:** Department of Veterinary Medicine, University of Sassari, Sassari, Italy; University of South Carolina, UNITED STATES

## Abstract

Roe deer (*Capreolus capreolus*) is the most abundant cervid in Europe and, as such, has a considerable impact over several human activities. Accurate roe deer population size estimates are useful to ensure their proper management. We tested 3 methods for estimating roe deer abundance (drive counts, pellet-group counts, and camera trapping) during two consecutive years (2012 and 2013) in the Apennines (Italy) in order to assess their precision and applicability. During the study period, population density estimates were: drive counts 21.89±12.74 roe deer/km^2^ and pellet-group counts 18.74±2.31 roe deer/km^2^ in 2012; drive counts 19.32±11.12 roe deer/km^2^ and camera trapping 29.05±7.48 roe deer/km^2^ in 2013. Precision of the density estimates differed widely among the 3 methods, with coefficients of variation ranging from 12% (pellet-group counts) to 58% (drive counts). Drive counts represented the most demanding method on account of the higher number of operators involved. Pellet-group counts yielded the most precise results and required a smaller number of operators, though the sampling effort was considerable. When compared to the other two methods, camera trapping resulted in an intermediate level of precision and required the lowest sampling effort. We also discussed field protocols of each method, considering that volunteers, rather than technicians, will more likely be appointed for these tasks in the near future. For this reason, we strongly suggest that for each method managers of population density monitoring projects take into account ease of use as well as the quality of the results obtained and the resources required.

## Introduction

In recent years, part of the scientific community in Europe raised doubts about the usefulness of methods for estimating large mammal population abundance in order to plan management options [1,2]. Indeed, assessing the number of individuals within a certain population is not enough to determine whether their pressure on the environment exceeds the limits that managers consider acceptable. Given the difficulty in obtaining a reliable population estimate in the wild and adopting a more ecosystem-oriented approach, Morellet [2] proposed to focus on and monitor the impact of ungulates on forests and agricultural lands and to use it as a local environment-specific indicator of population levels. Although this approach can be a valid alternative to population size estimates in areas where the focus is on controlling their impact on vegetation, it is less suitable for planning the management and conservation of large mammal populations [3]. On the other hand, population size estimate is generally considered a minimum requirement to plan wildlife management [4]. Moreover, understanding trends in population size can be useful to a sustainable management planning. In fact, rather than focusing on the mere number of individuals in a population, long-term monitoring programs should provide information on its trends, habitat requirements, the impacts of anthropogenic activities, and the damages that species caused to agriculture and forestry. In fact, the estimation of the damages, and the drivers affecting them, are particularly important in the case of species which, such as deer, have a remarkable economic impact on human societies [5].

As it is hardly feasible to count all the individuals in a wild population, sampling techniques are generally used to estimate their density in a given area [6]. A range of different techniques and methods for estimating wild ungulate population size are used in Europe and sometimes even within the same country according to different management objectives [7]. Such methods can be subdivided into two main categories: direct and indirect ones. Direct methods require the visual observation of individuals by operators, which can also provide information other than their number. For instance, operators can report on population structure (e.g., proportions of sex and age classes and group composition) and behaviour. However, these methods are demanding, time-consuming and therefore often restricted to specific sampling periods and areas [8,9]. The environment where the survey is performed and the consequent detectability of the species must always be taken into account when choosing the sampling method. In fact, direct methods were found to perform poorly on account of some species-environment combinations. For example, Andersen [10] found that roe deer (*Capreolus capreolus*) density estimated by direct observations and drive counts in broad-leaved woodland was one-third of the actual one. Bongi [11] found that, in a study in the Apennines, only ca. 70% of a tagged sample of individuals known to be in the drives were actually observed by operators. Moreover, these species-environment constraints can be a pivotal issue when target species have strong seasonal behaviour and distribution as well as when their abundance changes frequently and rapidly because of environmental conditions. Finally, the presence of the operator(s), if perceived, may alter the individuals’ behaviour (e.g., causing them to flee) and lead to biased results.

In response to these setbacks, indirect methods have been developed. They rely on such signs left by individuals as faeces [8], footprints, sounds, carcasses, scratches or other marks left on trees, all of which can be used to estimate their absolute or relative (i.e., compared to past estimates) abundance [6]. Indirect methods usually have fewer constraints, though they are also affected by behavioural and environmental conditions and usually cannot be used to collect information about population structure and vital rates. In the last few decades, several indirect methods have been proposed and tested in order to assess the abundance of populations through the analysis of persistent signs where the direct methods were found to fail. Even though the reliability of such indirect methods is not yet consensual [12,13], they are usually less demanding and time-consuming than direct methods. Moreover, given their lower disturbance, they also reduce the aforementioned bias caused by behavioural modifications. So far no method has proved perfectly accurate for each and every context. Still, decades of research have led to innovative and more robust methods, such as the capture-mark-recapture (or resight; e.g. MARK [14]). Nonetheless, most methods are either too complicated or costly to be used regularly in a management context and are therefore employed only in scientific research.

Our study focuses on roe deer, which is the most abundant and widely distributed ungulate in Europe and likely the species most frequently involved in count activities by wildlife managers in several European countries. Despite the development of new count techniques useful to estimate roe deer density, most managers use vantage points counts and drive counts to estimate their population size [7]. This is arguably out of habit, one which is made possible by the high number of hunters available to implement the counts. However, the negative trend of hunter populations in Europe urges an assessment of the pros and cons, of each count method, including economic and time demands, in order to define the relative best practices. We analysed three sampling methods: drive counts [10,15–18], pellet-group counts [19–23], and camera trapping ([24–26] but see [27,28]):

- In drive counts, an area with well-defined boundaries is driven by a line of beaters who move from one side of the area and drive individuals towards observers stationing along the remaining sides. The count is the number of individuals observed while leaving the area by either the drivers or the observers.
- Pellet-group counts assesses roe deer density by using the total number of pellet groups observed within the sample plots, considering the number of pellet groups deposited per deer per day, the period length and the area sampled.
- Random Encounter Method (REM) uses the ratio of photographs per day, while considering the real area surveyed by the traps (detection area) and the mobility rate of the species. The method, developed by Rowcliffe [29], analyses information from camera trapping without the need for individual recognition and overcomes the issues related with trapping rate biases by modelling the underlying dynamics of the encounters between individuals and cameras.

The goal of the study was to test the precision and the applicability of the three methods for roe deer in a broad-leaved forest of the Apennines. Moreover, we collected information about the requirements of each method, from planning to data collection, in order to assess their demands and whether these are justified by the quality of their results.

## Materials and methods

### Study area

**Fig 1:**
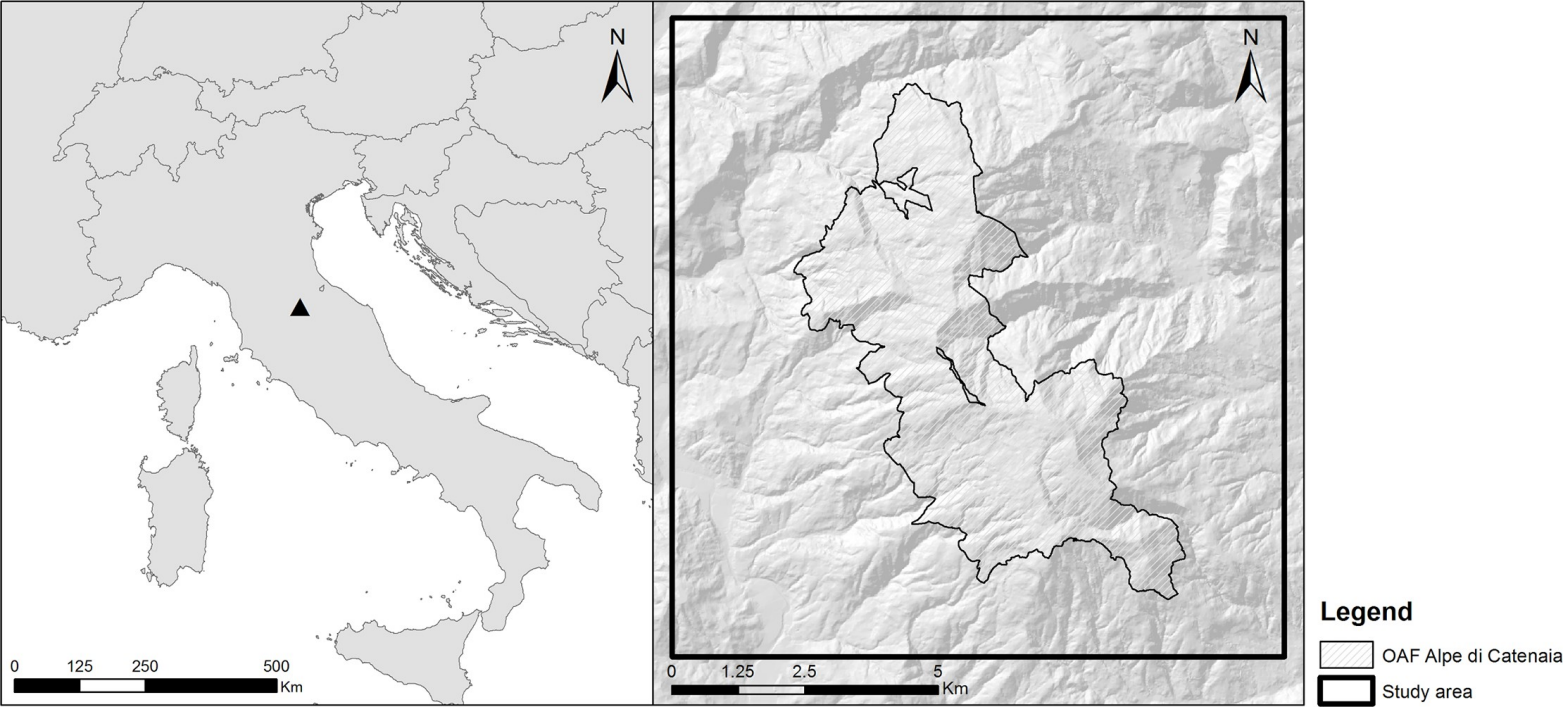
Location of the study area in the Italian Apennines and boundaries of the protected area it encompasses.

The study area encompasses 13,800 ha of the Casentino valley, Tuscany, Italy (43°39'36.2"N, 11°55'27.1"E). It is located in a mountainous region in the Northern Apennines and covers an altitude range between 248 and 1,414 m a.s.l.. The study area includes a protected area of 2,760 ha that lies at the highest altitudes (Fig 1). The climate is temperate (Cfc in Köppen’s classification), with hot and dry summers, cold and rainy winters, and a high humidity rate. Mean annual precipitations in the area range between 900 and 1,500 mm. Forests cover 67% of the area, while urban areas cover 4% of it. The rest is composed by agricultural areas (18%) and shrubs (11%). Forests mainly comprise broad-leaved species, the most abundant being the oak (*Quercus* spp.), the beech (*Fagus sylvatica*), the chestnut (*Castanea sativa*), and the hornbeam (*Ostrya carpinifolia*). The most common conifers (8% of the forested area) are the white fir (*Abies alba*), the black pine (*Pinus nigra*), the Douglas fir (*Pseudotsuga menziesii*), and the maritime pine (*Pinus pinaster*).

Four ungulate species are present in the study area: the roe deer, the wild boar (*Sus scrofa*), the fallow deer (*Dama dama*), and the red deer (*Cervus elaphus*). Roe deer and wild boar are abundant in the whole study area, while the densities of red deer and fallow deer are clearly lower, with a heterogeneous distribution. The wolf (*Canis lupus*) and the red fox (*Vulpes vulpes*) are the two main predators. Our study area encompasses part of the territory of at least two wolf packs (with a mean of 4 individuals per pack estimated by means of wolf howling surveys, unpublished data) for which ungulates are the main prey items [30]. Fox mainly preys on roe deer fawns [31]. Roe deer hunting by shooting from fixed high seats is reported outside the protected area (for more details, see [32,33]).

### Data collection and data analysis

Data were collected in two subsequent years, i.e., 2012 and 2013. Drive counts in our study area are performed yearly by the Province in association with Unione Regionale Cacciatori dell’Appennino (URCA, regional union of Apennines hunters). Hence, they were performed in both 2012 and 2013. As for the other two methods, their application depended on the availability of resources and workforce of our research group alone and, as a result, only one of them was used each year. This prevented the application of all three methods on the same population (i.e., as population size varies in time). However, since the aim of the study was to compare the precision and applicability of the three methods and not to assess their accuracy and relative performance, the issue of the implementation of different count activities in two consecutive years was supposed to be negligible.

#### Drive counts

We performed 15 drive counts within our study area between April and May, in 2012 and 2013, following the protocol described in [34]. Drive counts sites were designed in order to be representative of the whole study area and easily accessible by operators. The 15 sampling sites were the same during both years of data collection. During the counts, the distance between operators was such that no roe deer could be missed and depended on vegetation density and structure of the site. Areas surveyed by drive counts had an average extension of 34.92±6.79 ha and were stratified across vegetation categories in the area; the average effort for each sampling area involved 110 operators/ha [34]. The French National Institute for Environmental and Agricultural Science and Research (formerly C.E.M.A.G.R.E.F. and currently IRSTEA) indicated that only forested areas should be selected for this technique [15], though this might lead to biased estimates in a heterogeneous habitat. For example, if the drive counts site is the only forested patch in an otherwise extensive agricultural land, all roe deer that live in that area might be hiding inside it. In this case, if we considered that all the individuals detected in the forested patch only live there, our density estimates would be highly inflated. For this reason, we used the correction factor proposed by Davis [30] and took into account the percentage of forested area out of the whole area surrounding the drive counts site. We therefore calculated a 1 km buffer around the area being surveyed and used its percentage of forest cover as a correction factor for the density estimation of that particular drive counts site. This correction factor was applied simply by multiplying the percentage of forest cover of the buffered area by the density estimate of that sampling site. The overall density estimate was calculated as the average density over all the sampling sites within our study area [15].

#### Pellet-group counts

The pellet-group counts technique was performed between May and July 2012. We used the faecal accumulation rate method (FAR), which requires two surveys to each sampling plot [35–37]. During the first survey, operators cleaned the area of all pellet groups, while during the second survey they counted all the pellet groups dropped in the meantime. This technique requires choosing the appropriate time span between the two surveys, which must be long enough to allow individuals to visit the area and short enough to be certain that no pellet group has decomposed and will not be counted. A decay rate analysis performed in our study area in late-spring/early-summer showed that roe deer drops completely decompose after a minimum of 28 days (Donaggio E., personal communication). Therefore, we opted for a time span of 21 days between the first and second survey. Roe deer mean defecation rate was estimated following Mitchell et al. [38], who observed a daily defecation rate between 17 and 23 pellet groups, based on the productivity of the environment; accordingly, we decided to use the mean value of 20 pellet groups per day. We used the mean value instead of the range as our aim is to assess the method precision, and not its accuracy.

Sampling design and the subsequent analysis were performed following the methodology described by Fattorini [39]. We assumed that roe deer density would differ between habitats. Therefore, starting from CORINE Land Cover dataset (2006), we grouped land use categories to obtain sets of similar habitats (Table 1). As Sutherland [40] stated, one of the most common mistakes in estimating a species’ abundance is to sample few large areas instead of several small ones. For this reason, we clipped the CORINE map by overlaying a 500 m grid, so that the widest land use polygon would be 25 ha. Moreover, we stratified the sampling plots between habitat sets, based on their extent (Table 1). The land use polygons to sample were chosen randomly, with a probability of being extracted proportional to their extent (i.e., wider polygons had a higher probability of being selected). This step was performed because, in the subsequent analysis, the data collected in each of the polygons selected for sampling would contribute to the density calculation proportionally to the polygon extent, i.e., wider polygons would weigh more in density calculations [39]. For a better density estimate of the polygon selected, 5 sampling plots were located within each of them. Instead of randomly placing the 5 plots within the polygons, we divided each polygon into 5 areas of equal extent and similar shape, and then randomly placed a single sampling plot within each of these areas. The splitting procedure was performed by using Brus algorithm, implemented in an R procedure [41]. This allowed us to maximize the representativeness of the results for the sampling area–i.e. the land use polygon–[42]. This protocol of data collection led us to a theoretical sampling effort of 300 plots. The plots were displaced by using a GIS software (ArcGIS 10.0, E.S.R.I.) and located on the field with a handheld GPS device. Six out of the 300 plots could not be reached by operators and, therefore, the plots monitored were 294. Each plot consisted of a round area with a radius of 5 m and a surface of 78.54 m^2^ each. A pellet group was defined as a group of at least 10 faecal pellets and two pellet groups were considered as distinct when separated by at least 1m [19]. The method developed by Fattorini [39] yields an estimate of the number of pellet groups present during the monitored time span for each of the strata considered in the sampling design. The sum of these estimates, divided by the time span, the defecation rate of the species, and the extent of the study area, provides the density estimates for the area.

**Table 1:** List of the vegetation strata considered with their size in hectares, in percentage with respect to the total area, and the number of polygons and sampling plots for each stratum in the study area of roe deer surveys in the Italian Apennines.

Stratum	Area (ha)	Area %	Number of polygons	Number of plots
Coppice	3818.5	28.8	17	85
Beech wood	2741.1	20.7	12	60
Oak wood-Chestnut wood	1973.9	14.9	9	45
Conifers	755.1	5.7	4	20
Shrubs	1525.0	11.5	7	35
Agricultural	2451.2	18.5	11	55
Total	13264.7	100	60	300

#### Random encounter method

Random Encounter Method was performed between April and May 2013. This method requires the encounters between individuals and cameras to occur randomly, and several sampling designs meet this assumption [43]. We stratified our camera locations over the same vegetation categories used for pellet-group counts and deployed our cameras randomly in 60 locations. The minimum distance between locations was set at 500 m so as to minimize the risk of spatial correlation between closely located cameras. We used 15 cameras: 9 MultiPR-12 and 6 Bushnell HD. Cameras were swapped and used in different locations. Each location was monitored for 2 weeks. Locations were identified by means of a handheld GPS device and cameras were set on trees and bushes at an approximate height of 50 cm and pointing toward North so as to minimize the interference of direct sunlight. This height was chosen so that ground vegetation would not interfere with camera performance and any roe deer passing by (the average shoulder height of roe deer captured in the area being 72 cm) may not be missed. Moreover, since the vegetation was very thick in a number of locations, we positioned the cameras in sites with vegetation-free space in front of them.

An individual or a group may trigger the camera several times in a row by walking slowly, lingering in the area, or simply owing to their high number. In such cases, all the pictures taken belong to a single-encounter event between the camera and either the individual or the group. A number of authors set a delay after the triggering of the camera and before another picture can be taken to prevent multi-triggering for a single-encounter event [29,44,45]. However, we did not set any delay after a picture was taken and identified the independent encounter events by inspecting all the pictures. As we applied a stratified sampling, we calculated the density estimates for each vegetation stratum considered. Then, the overall density estimate was obtained as the average of the per-stratum estimates weighed on the stratum surface with respect to the whole area. The formula used to calculate the density estimate in each stratum is the following [29]:

$$D=\frac{\frac{y}{t}\cdot \pi }{vr(2/\theta )}$$ (Eq 1)

The *y/t* variable is the trap rate, which is the number of independent events of encounter of a species divided by the days of monitoring effort (i.e., trap-days); *v* is the average daily speed of the target species; *r* and θ are the detection area radius and angle of the camera. The average daily speed of roe deer was found to be 1 km/day in the literature [46]. According to the technical specifications of the cameras, the radius and the angle of the detection area were 20 m and 80° for the Multi-PIR12 model, and 15 m and 50° for the Bushnell model. To obtain single values for each parameter to use in the REM model, we calculated the average of both detection area radius and angle of the cameras by using the number of locations where each camera model was used as weight. This calculation was performed for every stratum, as different combinations of the two camera models were used in each of them, resulting in different detection area parameters to use for each per-stratum density estimate calculation (Table 2).

Roe deer social structure varies over the year, with individuals being solitary and territorial in spring and summer, and joining social groups in winter [47]. For this reason, we used random encounters data collected in the same months of the monitoring effort (i.e., April and May) during research activities carried out in the area between 2008 and 2012 (n = 208), in order to calculate an independent estimate of the mean number of individuals in a group, which resulted to be 1.22. This value was multiplied by the result of the Eq 1 for each stratum to account for the independent encounter events caused by roe deer groups and not by individuals.

**Table 2:** Number of trap-sites, detection area (D.A.) angle and radius, trap-rates, and density estimates for each stratum, used for the calculation of roe deer density estimated by REM in the Italian Apennines.

Stratum	Trap-sites	D.A. angle	D.A. radius (km)	Trap-rate (pics/days)	Density Estim. (ind/km^2^)
Coppice	17	65.89°	0.017	0.182	12.56
Beechwood	11	58.16°	0.016	0.333	25.88
Oak-Chestnutwood	9	59.99°	0.017	0.416	31.38
Conifers	4	50.02°	0.015	0.269	23.93
Shrubs	7	58.56°	0.016	0.362	27.92
Agricultural	11	66.35°	0.018	0.858	58.72

#### Coefficient of variation and 95% confidence interval estimates

Coefficient of Variation (CV) was calculated for all methods as the ratio between the estimated density standard deviation and the estimated density itself: CV = sd/D, where D is the density estimation and sd is its standard deviation. The 95% Confidence Interval (CI) for the estimation was calculated for all methods as CI = D ± 1.96*sd, where D is the density estimation and sd is its standard deviation.

As for drive counts, standard deviation was calculated from the weighed density estimations of each of them (see *Drive counts* paragraph). As for the pellet-group counts, standard deviation was calculated as the square root of the variance of the estimator used to estimate the number of pellet groups in each stratum ([48], see Pellet Group Count Paragraph and [39]). Since the REM model does not return an estimate of the error of the result, following Rowcliffe [29], we used non-parametric bootstrap on camera locations with 10,000 replicates to estimate the standard error. Our sampling design was stratified by vegetation categories, with a different number of trap-sites for each stratum. The replacement of trap-sites during the resampling procedure (bootstrap) occurred within each stratum in order to maintain the same proportion of traps among the vegetation categories.

#### Effort required

The effort required was estimated by recording the number of operators and the time needed to complete each phase of the three methods, i.e., sampling design, field data collection, data entry and data analysis. We did not provide an estimation of the cost since this varies among the countries and according to the year.

On the basis of Italian National law (n. 157/1992), local bodies (Regional and Provincial governments) are responsible for the wild fauna's welfare and management in Italy. The drive counts are implemented and managed by the Province of Arezzo. The overall research project was formally approved by Tuscany Regional Administration (n. 103/5936/152–13/03/2002), even if the study did not involve capture or manipulation of wild animals.

## Results

### Drive counts

**Fig 2:**
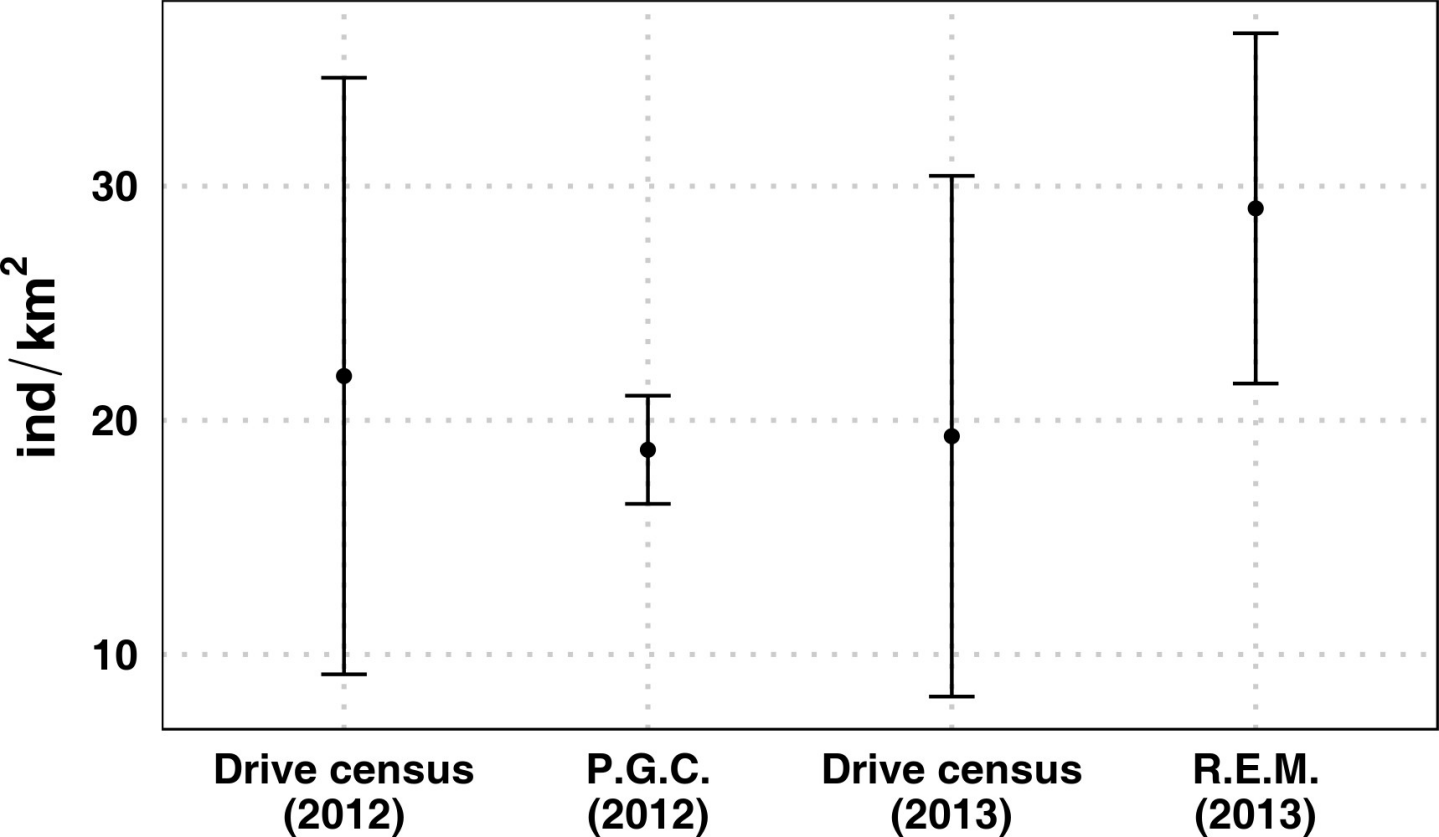
Density estimates and standard deviations for each method considered. Parentheses indicate the year when the sampling was performed. P.G.C. stands for pellet-group counts.

The mean extension of drive counts sites was 34.92 ± 6.79 ha, covering a total extent of 523.80 ha. The density estimates for the single site ranged between 6.81 and 46.14 individuals/ km^2^ in 2012, and between 6.34 and 45.20 in 2013. Following C.E.M.A.G.R.E.F. [15], the density estimate for the whole study area was calculated as the average density of the drives performed. This led to an estimated population size of 21.89±12.74 and 19.32±11.12 individuals/ km^2^ in 2012 and 2013, respectively (Fig 2).

#### Pellet-group counts

The number of pellet groups found in a single plot ranged from 0 to 7, with a frequency distribution markedly shifted towards the lower end of the range (Fig A in S1 File). In our study area, considering a daily defecation rate of 20 pellet groups, density estimate resulted to be 18.74 ± 2.31 individuals/ km^2^ (Fig 2).

#### Random encounter method

We employed cameras for a total of 765 trap-days, during which they recorded 2,098 pictures of individuals of different species. Since one camera did not work, one of the locations could not be included in our analysis (n sites = 59). Roe deer was the second most frequently recorded species, with 850 pictures which, once visually analysed, identified 294 independent encounters. Trap-rates and average detection area parameters for each stratum are reported in Table 2. The overall density estimate was calculated as the weighed average of per-stratum REM density estimates, setting a daily speed of 1 km/day, and resulted to be 29.05 ± 7.48 individuals/ km^2^ (Fig 2).

#### Estimation precision

The density value estimated by means of drive counts for the year 2012 is 21.89 ± 12.74 ind/km^2^, with a CV of 58% and a 95% CI spanning from 0 to 46.87 ind/km^2^. The density estimate for 2013 is 19.32 ± 11.12 ind/km^2^, with a CV of 58% and a 95% CI spanning from 0 to 43.72 ind/km^2^ (Table A in S1 File). Interestingly, the CV value of the estimated population size is the same in both years.

The density estimate returned by pellet-group counts for the year 2012 is 18.74 ± 2.31 ind/km^2^, with a CV of 12% and a 95% CI spanning from 14.21 to 23.27 ind/km^2^ (Table A in S1 File). The density estimate returned by REM for the year 2013 is 29.05 ± 7.48 ind/km^2^, with a CV of 27% and a 95% CI spanning from 14.39 to 43.72 ind/km^2^.

Drive counts showed the lowest precision (CV: 58%), REM performed better (CV: 27%), and the pellet-group counts showed the highest precision (CV: 12%).

When we compared the density estimates in the same year, i.e., drive counts and pellet-group counts in 2012 and drive counts and REM in 2013, the density estimate of each method contained the other estimate in its 95% CI.

#### Effort required

As for drive counts, one operator worked for a week to select the sampling sites, while two operators worked for two weeks to prepare them, i.e., to mark their perimeters and make them easily identifiable. This technique required the presence of at least 90 operators for 5 days of fieldwork during the counts. After the counts, a single operator recorded the data in one day. Moreover, the coordination of all the operators required an intense organizational effort, which should not be disregarded. We estimated an organizational work of at least 7 days carried out by one person which corresponds to the work of several staff members of both the Province and the URCA who usually organize the fieldwork and get a high number of hunters involved. We assessed a total effort of 3,564 working hours, mainly concentrated in the days of the drives, when the presence of operators was necessarily massive (70–90 people). On the other hand, the count activity required a relatively short fieldwork of approximately 5 days (Table 3).

**Table 3:** Number of sampling areas, total extent sampled, number of operators and time required for fieldwork, number of operators, and time required for data entry, total time effort required, and precision of the method expressed as coefficient of variation (CV).

Method	Sampling areas	Total sampled extent (ha)	Operators required	Field effort	Data entry effort [operators]	Estimated effort hours	Precision (as CV)
Drive counts	15	523.80	70 ~ 90	5 days	1 day [1]	3564	58%
Pellet-group counts	294	2.31	4	2 months, 5 days a week	2 days [1]	2936	12%
R.E.M.	60	0.016	2	5 days every other week for 2 months,	10 days [2]	496	27%

The displacement of the plots for the pellet-group counts by using the GIS software required one week of work by a single operator for the design-based sampling used. The method did not require any preliminary field preparation, whereas the count itself needed 4 operators working for 2 months. After the sampling, one operator can perform data entry in 2 days’ work. Although data collection was longer (2 months), it involved a lower number of operators (4 people) and the whole effort was partially lower (2,936 working hours) in comparison to drive counts (Table 3).

Camera-trap sites displacement required one operator working for 2 days on a GIS. Once the locations were selected, the count could start without additional fieldwork. The time span of camera-traps deployment varies on the basis of the target species and environmental characteristics. In our case study, it required 2 operators working every other week for 2 months. Data entry required more time if compared to the other methods, as all the pictures recorded had to be inspected in order to identify encounter events and this value is subject to case-specific variations due to the individual’s abundance, behaviour and environmental characteristics. For our study, it required 2 operators working for 10 days. This technique required the lowest effort (496 work hours) and the lowest number of operators (2 people). However, the period of field data collection was long (2 months) and the data entry effort was considerable (10 days; Table 3).

## Discussion

Our results show that the three methods employed yielded relatively consistent estimates of a roe deer population, which may suggest a coherence among the methods. However, estimates precision as well as the efforts required to implement each method differed notably. Pellet-group and drive counts showed the highest and lowest precision, respectively, while REM presented an intermediate level of precision. Although limited to a single study area, these findings seem to support the use of pellet-group counts for roe deer in forested study areas, as it proved the most precise method.

Drive counts proved the least precise method (C.V. = 58.0% in both years, Table A in S1 File) owing to the high variability in individual counts among the different areas surveyed. Several factors may have affected the operators’ counting performance, such as weather conditions, vegetation closeness and thickness, different levels of noise during the operators’ disposition prior to the drive, and the time of the day when the drive was performed (e.g. [11]). On the other hand, since sampling sites were designed in order to be representative of the whole study area, the difference in roe deer numbers counted among drives may be a genuine indication of the heterogeneous spatial distribution of the species.

The pellet-group counts method returned the most precise results, with a CV of 12.0% (Table A in S1 File). The density estimate given by pellet-group counts is slightly lower than that obtained by drive counts. This difference may be accounted for by the failure in finding all the pellet groups present in a plot: for instance, pronounced slopes and dense ground vegetation areas [49] may have hindered the detection of all the pellet groups by the operators.

The REM provided a higher density estimate than drive counts for year 2013 with a CV of 27.0%. However, some doubts might be raised about the parameters of the cameras’ detection area and the species’ daily speed used to calculate our estimates. We used two different camera models and calculated the detection area parameters as a weighed average of the values reported in the technical specification of each model. We acknowledge this as a potential source of bias for our density estimate. Cusack [45] performed a sensitivity analysis of the effect of the variation of cameras’ detection area on the density estimate and found that a 1% variation of detection radius and angle values resulted in a change in density of 1% and 0.3%, respectively. According to Cusack, our density estimate may show a 13–15% bias owing to our averaging of the data from the two camera models. The other source of bias may be the average daily speed of the species, which in other studies resulted to be the most sensitive parameter [50,51]. Given the lack of roe deer fine movement data in our study area, we extracted the value from the literature [46]. Since the value is provided with no error estimation, it was not possible to insert its uncertainty in the variance calculation of the final density estimate. Our aim was to compare the applicability and the effort required amongst these widely used density estimation methods. Therefore, although we acknowledge the potential sources of bias inherent to our application of the REM, given that more recent protocols for its application yield better results (i.e., with less biases) and required lower effort, we believe that our application is nonetheless robust in the context of this paper.

Management practices often have to compromise between the quality of their results and the effort required to obtain them. The methods we tested differ notably across the effort they require in terms of both time and workforce. Drive counts require the highest number of operators, who need to hedge the sampling area and maintain visual contact among them. The organization of such a crowded event is not easy and the suitable days to perform the drives and the number of drives that can be performed in a single day are limited by the availability of operators. The preparation of 15 drive areas requires at least 2 operators working for a couple of weeks, as the perimeter of each area has to be marked with labels indicating the position of each operator and sometimes the vegetation along that perimeter has to be reduced in order to allow for clear sighting between operators during the count. The pellet-group counts method has lower workforce requirements (4 operators were necessary to perform our sampling protocol), but it is time consuming, as visiting ca. 300 plots twice may take as much as 2 months of work. Moreover, the sampling design and the subsequent data analysis are quite complex and may need the help of a statistician. The fieldwork of REM requires the lowest number of operators (only 2), although, on the subsequent stage, the time needed to inspect all the pictures strongly depends on the number of camera-sites used and, of course, on the total number of pictures recorded by the cameras. The latter usually depends on the vegetation in the site and the weather conditions (e.g., wind). Then, to select and analyse the pictures takes several days. On the other hand, software development is running fast to support researchers and managers with programs that automatize most of the work necessary to record pictures’ data and are able to easily manage the vast amount of data collected by either camera- or video-trapping (e.g. [52,53]). Both sampling design and the subsequent analysis can be easily performed, but the method requires a considerable economic investment for the cameras, depending on the models and number. In our effort estimation, we did not consider the statistical analysis that follow data entry, as we took it for granted that an expert technician or analyst needs about the same time to process the data for all three methods. Moreover, an expert in statistical analysis might be necessary to process the data—at least for pellet-group counts—in order to obtain density estimates. And this cost represents an additional economic demand of this method.

In conclusion, our estimations of the effort required showed a marked difference among the methods in their time requirements. Drive counts and pellet-group counts require between 3,000 and 3,500 working hours, while REM required less than 500, i.e., between 14% and 17% ca. of the working hours needed by the other two methods (Table 3). This might be important from a management viewpoint, since it both reduces the costs of the survey and allows for the repetition of the sampling throughout the year, which may be useful in the case of a species with strong seasonal variation or for a finer population dynamic analysis. Another issue that has to be considered when choosing a sampling method is the operators’ training. On the one hand, it may be difficult to reach the number of operators needed for such a method as drive counts; on the other hand, if the method requires specialized operators, their hiring may be a considerable economic effort. In addition, it is worth noting that drive counts in Europe are widely performed with the help of hunters, who are decreasing and aging (e.g. [54]), and this will likely increase the difficulty of implementing surveys requiring many people.

Lastly, as the density estimates of all methods are congruous, the choice of the method seems to depend on logistic issues as well as on the human and economic resources of each study area. The implementation of these methods gives adequate results only if their protocols are properly followed and their assumptions met. In this respect, as in the next future the performing agents will likely not be technicians on account of the widespread budget cuts in wildlife management, the ease of fieldwork will be an important issue.

Nonetheless, to achieve reliable results, the methods must comply with adequate knowledge of a number of species and site-related parameters such as daily defecation rate and daily range. These data should be acquired by means of appropriate studies performed by technicians and researchers. Some of these studies may be quite demanding and expensive, though, fortunately, they do not need to be repeated every time the counts are performed. In light of the recent trends in hunters and volunteers populations and the increasing complexity of the methods developed, we wish to stress the importance of comparative studies on density estimation techniques, which should not only test the quality of the results, but also assess their efforts and costs, and, finally, their possibility to be implemented by non-technicians. As studies on population dynamics usually cover a long time span, it is important to consider the requirements of each method to be able to choose a technique which can be applied in the long run, with an allegedly limited number of qualified operators.

## Supporting information

S1 File Graphic and table supporting the results of the paper. Fig A Distribution of the number of pellet-groups found for each plot during the count. For more details about the definition of pellet-group and methods of data collection, see the text. Table A. Density estimates with standard deviation, coefficient of variation (CV) and 95% confidence interval (CI) for each method considered for roe deer density estimation.
(DOCX)
