# Supplementary material for: Assessing precision and requirements of three methods to estimate roe deer density
Source: PLoS One. 2019 Oct 10;14(10):e0222349. doi: 10.1371/journal.pone.0222349 (PMC6786588; doi:10.1371/journal.pone.0222349)
Supplement: S1 File — Fig A Distribution of the number of pellet-groups found for each plot during the count. For more details about the definition of pellet-group and methods of data collection, see the text. Table A. Density estimates with standard deviation, coefficient of variation (CV) and 95% confidence interval (CI) for each method considered for roe deer density estimation. (DOCX) [file pone.0222349.s001.docx]

**Supplementary materials**

**Fig. A** Distribution of the number of pellet-groups found for each plot during the pellet-group count. For more details about the definition of pellet-group and methods of data collection, see the text.


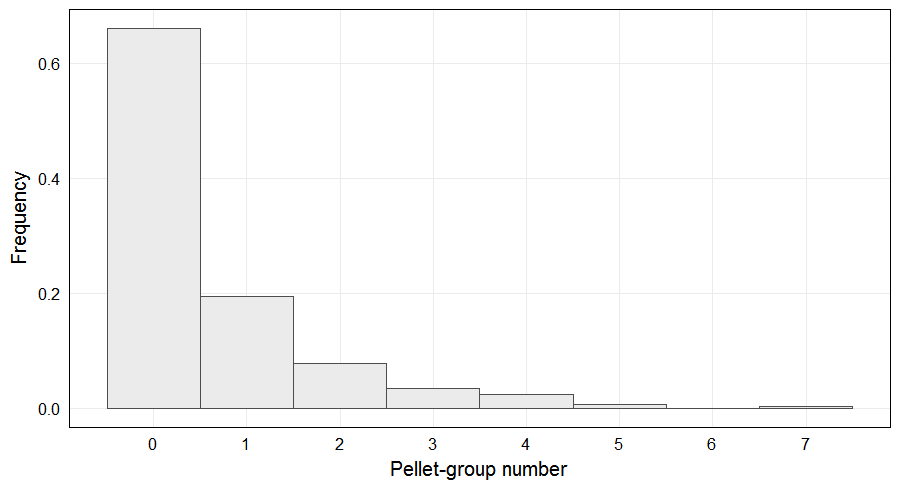


**Table A** Density estimates with standard deviation, coefficient of variation (CV) and 95% confidence interval (CI) for each method considered for roe deer density estimation in the Italian Apennines.

| **Method** | **Density (km^2^)** | **Standard Deviation** | **C.V.** | **95% C.I.** |
| --- | --- | --- | --- | --- |
| Drive census 2012 | 21.89 | 12.74 | 58% | 46.87-0.0 |
| Drive census 2013 | 19.32 | 11.12 | 58% | 41.11-0.0 |
| Pellet-group count | 18.74 | 2.31 | 12% | 23.27-14.21 |
| R.E.M. | 29.05 | 7.48 | 27% | 43.72-14.39 |
